# Effects of fetal exposure to high-fat diet or maternal hyperglycemia on L-arginine and nitric oxide metabolism in lung

**DOI:** 10.1038/nutd.2016.56

**Published:** 2017-02-20

**Authors:** C Grasemann, R Herrmann, J Starschinova, M Gertsen, M R Palmert, H Grasemann

**Affiliations:** 1 Pediatric Endocrinology and Diabetology, Kinderklinik II, Universitätsklinikum Essen and University of Duisburg-Essen, Essen, Germany; 2 Division of Neonatology, Kinderklinik I, Universitätsklinikum Essen and University of Duisburg-Essen, Essen, Germany; 3 Division of Pediatric Endocrinology, Department of Pediatrics, The Hospital For Sick Children, Toronto, ON, Canada; 4 Department of Physiology, The University of Toronto, Toronto, ON, Canada; 5 Division of Respiratory Medicine, Department of Pediatrics, The Hospital For Sick Children, Toronto, ON, Canada; 6 Program in Physiology and Experimental Medicine, Research Institute, The Hospital for Sick Children, Toronto, ON, Canada

## Abstract

**Background/Objectives:**

Alterations in the L-arginine/nitric oxide (NO) metabolism contribute to diseases such as obesity, metabolic syndrome and airway dysfunction. The impact of early-life exposures on the L-arginine/NO metabolism in lung later in life is not well understood. The objective of this work was to study the effects of intrauterine exposures to maternal hyperglycemia and high-fat diet (HFD) on pulmonary L-arginine/NO metabolism in mice.

**Methods:**

We used two murine models of intrauterine exposures to maternal (a) hyperglycemia and (b) HFD to study the effects of these exposures on the L-arginine/NO metabolism in lung in normal chow-fed offspring.

**Results:**

Both intrauterine exposures resulted in NO deficiency in the lung of the offspring at 6 weeks of age. However, each of the exposures leading to different metabolic phenotypes caused a distinct alteration in the L-arginine/NO metabolism. Maternal hyperglycemia leading to impaired glucose tolerance but no obesity in the offspring resulted in increased levels of asymmetric dimethylarginine and impairment of NO synthases. Although maternal HFD led to obesity without impairment in glucose tolerance in the offspring, it resulted in increased expression and activity of arginase in the lung of the normal chow-fed offspring.

**Conclusions:**

These data suggest that maternal hyperglycemia and HFD can cause alterations in the pulmonary L-arginine/NO metabolism in offspring.

## Introduction

Nitric oxide (NO) and NO metabolites are messenger molecules that are involved in a number of physiological and pathophysiological conditions, including obesity and metabolic syndrome.^1^ NO is the product of NO synthases (NOSs), which form NO and L-citrulline from the semi-essential amino acid L-arginine. All three isoforms of NOS are expressed in the human lung, but differ in their regulation and NO production. The availability of L-arginine for NOS is in part regulated by the enzyme arginase, which catalyzes the hydrolysis of L-arginine to urea and L-ornithine. Arginase exists in two isoforms, arginase 1 and arginase 2. Both are expressed in multiple tissues, including the lung.^2^ NO production from NOS can also be limited by presence of asymmetric dimethylarginine (ADMA), a product of protein degradation that acts as an NOS inhibitor. Increased ADMA was recently found in obesity, insulin resistance and Type 2 diabetes^3, 4^ and also in asthma.^5, 6, 7, 8^

Nutrition in early life plays an important role in predisposing an individual to the development of chronic diseases, including cardiovascular, metabolic and allergic conditions, later in life.^9^ The effects of hyper-alimentation during pregnancy on pulmonary metabolism in offspring are not well understood. We hypothesized that intrauterine exposures to maternal high-fat diet (HFD) or hyperglycemia would lead to changes in the L-arginine/NO metabolism in lung later in life.

## Materials and methods

To study the effects of *in-utero* exposures to maternal HFD and maternal hyperglycemia in mice, male offspring of wild-type mothers exposed to HFD and male wild-type offspring of hyperglycemic Akita mice (see details below) were studied. All experiments were approved by the Sickkids Research Institute Animal Care Committee.

### Mouse models of intrauterine exposures

#### Maternal hypoinsulinemic hyperglycemia

Akita mice (C57Bl6/J-Ins2<Akita>) harbor a heterozygous point mutation in the insulin 2 gene, which results in progressive hyperglycemia from 5 weeks of age.^10^ Heterozygous females with increased fasting glucose prior to mating and during pregnancy were bred to wild-type C57Bl6/J males, as described previously.^11^ Male wild-type offspring (50%) of these matings, which were exposed to maternal hyperglycemia *in utero*, developed impaired glucose tolerance at 14 and 26 weeks of age, despite normal fasted insulin levels, and reduced body weight.^11^

#### Maternal HFD

In this model of intrauterine exposure to maternal HFD, wild-type C57Bl6/J females were fed a diet with 45% kcal per fat (TD 06415, Harlan Teklad, Indianapolis, IN, USA) starting from 6 weeks prior to mating throughout pregnancy and lactation, as previously described.^12^ Offspring were weaned to normal chow (Harlan Teklad Global 2018, 18% kcal per fat) at day 21 of life and compared with offspring from control breedings. Male offspring showed increased weight at 14 weeks and decreased weight at 26 weeks of age. Glucose tolerance was normal at 14 weeks and only mildly impaired at 26 weeks of age.^12^

To test our hypothesis that intrauterine exposures to maternal hyperglycemia or HFD would affect pulmonary L-arginine/NO metabolism, we quantified the L-arginine metabolism in tissues of 6- and 14-weeks-old male wild-type offspring from both models.

Results were expressed as the mean±s.e.m. Binary comparisons were made with two-tailed Student's *t*-test or Mann–Whitney test, where appropriate. Statistical analysis was performed using GraphPad Prism 5 (La Jolla, CA, USA). Significance for all tests was defined at *P*<0.05.

## Results

### Offspring of both HFD and hyperglycemic mothers are NO-deficient at 6 weeks of age

**Figure 1:**
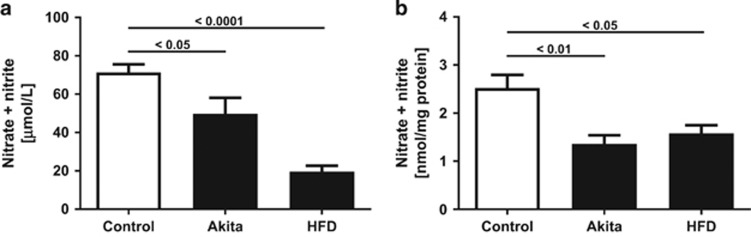
Effect of intrauterine exposures on nitric oxide concentrations. NO metabolite (nitrate plus nitrite) concentrations in (**a**) serum and (**b**) lung homogenates of 6-weeks-old male wild-type offspring are significantly reduced following intrauterine exposure to maternal hypoinsulinemic hyperglycemia (Akita) or maternal HFD, compared with normal control, respectively.

The NO metabolites nitrate plus nitrite were measured using the Griess reagent. Serum nitrate plus nitrite at 6 weeks of age was significantly lower in HFD and to a lesser degree also in Akita offspring compared with control (Figure 1a). A significant reduction in nitrate plus nitrite was also seen in lung homogenates of HFD and Akita offspring at 6 weeks of age (Figure 1b). Serum nitrate plus nitrite in 14-weeks-old wild-type offspring did not differ between controls, HFD and Akita offspring, and was similar to previously reported adult mice.^13^ Because of limited tissue availability, lung nitrate plus nitrite at 14 weeks could not be measured.

### Changes in the L-arginine metabolism in lung are specific to the dietary exposure *in utero*

**Figure 2:**
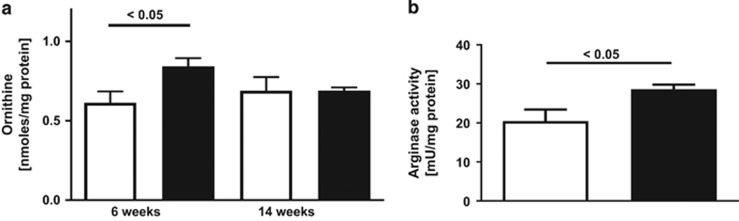
Effect of intrauterine exposure to HFD on pulmonary arginase activity. (**a**) Concentrations of L-ornithine, the product of arginase activity, are increased in the lung at 6 weeks but not in 14-weeks-old male wild-type offspring exposed to maternal HFD (black bars), compared with control (white bars). (**b**) Arginase activity in lung was also increased at 6 weeks of age.

While nitrate plus nitrite was significantly reduced in the lungs of both HFD and Akita offspring at 6 weeks of age, the two intrauterine exposures resulted in distinct effects on the pulmonary L-arginine and ADMA metabolism in the offspring. In male offspring of HFD mothers, the lung content of L-ornithine, a product of arginase activity, was significantly increased (*P*=0.037) at 6 weeks (Figure 2a). There was also a 1.6-fold increase in the lung expression of arginase 1 (*P*=0.03) but not arginase 2, as measured by rtPCR, and increased (*P*=0.0337, *n*=5 vs 5/group) total arginase activity, as measured by an *in vitro* assay,^14^ in the lungs of 6-weeks-old HFD offspring compared with control (Figure 2b). The lung content of L-arginine, and the methylated arginine derivatives ADMA, symmetric dimethylarginine and monomethyl arginine, which were quantified by liquid chromatography-tandem mass spectrometry (LC-MS/MS),^5^ did not differ between HFD offspring and controls at 6 or 14 weeks. However, there was a trend toward decreased L-arginine /ADMA, at 6 weeks in HFD offspring (262±34.5 vs 173±14.7, *P*=0.065) (*n*=7 vs 5/group). The L-arginine/ADMA ratio is an established marker of NOS impairment, and decreased ratios are associated with NO deficiency.^6, 15^

**Figure 3:**
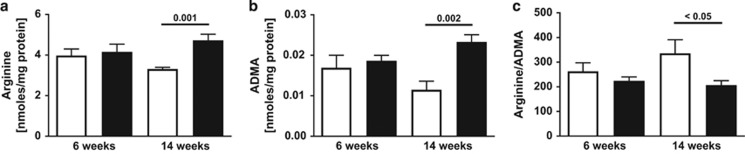
Effect of intrauterine exposure to hyperglycemia on pulmonary L-arginine/ADMA. Lung contents of (**a**) L-arginine and (**b**) ADMA are increased, while (**c**) the L-arginine/ADMA ratio is decreased in the lung of 14-weeks-old male wild-type offspring of hypoinsulinemic hyperglycemic mothers (Akita model; black bars), compared with control (white bars), respectively.

In contrast to HFD, no evidence for upregulated arginase or increased L-ornithine levels was present in the lung of the male offspring of hyperglycemic mothers. However, the lung content of ADMA, which was not different between Akita offspring and controls at 6 weeks, was increased (*P*=0.002) in Akita offspring at 14 weeks of age. Similarly, lung L-arginine levels were not different at 6 weeks but increased (*P*=0.01) at 14 weeks, as was symmetric dimethylarginine (*P*=0.002) and monomethyl arginine (*P*=0.0033). The L-arginine/ADMA ratio was decreased (*P*<0.05) at 14 weeks of age (Figures 3a–c).

To further elucidate the effect of intrauterine exposure to maternal hyperglycemia on ADMA metabolism in offspring, we studied the expression of protein arginine methyltransferases (PRMTs) and dimethylarginine dimethylaminohydrolase (DDAH1 and 2) in metabolic tissues by rt-PCR. PRMTs methylate protein arginine residues to form ADMA, symmetric dimethylarginine and monomethyl arginine, while DDAH degrades ADMA. The expression of PRMT1 was increased 1.4-fold (*P*=0.012) in the liver of Akita offspring at 6 weeks (*n*=15 vs 9/group), and was not different at 14 weeks (*n*=7 vs 4/group). DDAH1 in kidney showed a trend toward decreased (*P*=0.07, *n*=8 vs 5/group) expression in Akita offspring at 6 weeks, while DDAH2 was not different from control kidney.

## Discussion

Our data show that *in-utero* exposures to both maternal hyperglycemia and HFD can cause changes in the pulmonary L-arginine metabolism in male wild-type offspring. Specifically, we observed that maternal hyperglycemia leading to impaired glucose tolerance in offspring at 14 weeks of age resulted in an increase in lung ADMA content as well as NOS impairment (decreased L-arginine/ADMA ratio). No systemic NO (nitrate plus nitrite) deficiency was observed at this time point. However, both serum and lung nitrate plus nitrite levels were significantly reduced at 6 weeks of age in the animals, when ADMA and L-arginine/ADMA ratio were normal. Although this seemed counterintuitive, we did find early evidence for alterations in the ADMA metabolism in these animals at 6 weeks of life. Specifically, there was a significant increase in the expression of liver PRMT1, an enzyme that produces ADMA. In addition, the expression of the ADMA-metabolizing enzyme DDAH1 in kidney was lower in 6-weeks-old Akita offspring compared with control, although, likely due to low numbers of tissues studied, this difference between groups did not reach statistical significance. Our observations may suggest epigenetic regulations of the ADMA metabolism in this model or possibly modulations related to a glycosylation event.^16^ These findings add to previously reported work suggesting a link between maternal and fetal nitrogen oxide metabolism and disease.^17, 18^

In our second model, the intrauterine exposure to maternal HFD, leading to increased weight in the offspring but normal glucose tolerance at 6 and 14 weeks of age, resulted in a significant increase in arginase expression and activity in lung. As expected, the increase in arginase at 6 weeks of age was associated with decreased nitrate plus nitrite in lung as well as blood. Thus, both intrauterine exposure models, leading to distinct metabolic phenotypes, respectively (impaired glucose tolerance but no obesity, and obesity but no impaired glucose tolerance), cause specific but distinct changes in the L-arginine/NO metabolism, despite normal diet after weaning.

Previous studies had demonstrated a link between hyper-alimentation and airway dysfunction in older animals,^19, 20^ and 18 weeks of hyper-alimentation started at 6 weeks of age induced alterations in the pulmonary L-arginine/NO metabolism and airway dysfunction characteristic for asthma.^1^ Similar animal models revealed a strong association of maternal diet with fetal lung development,^21^ and also with airway inflammation and hyper-reactivity in offspring.^22^ Our studies add to these previous findings by showing that intrauterine exposures to maternal hyper-alimentation can induce specific changes in the pulmonary L-arginine/NO metabolism in offspring previously described to be characteristic for asthma,^5, 6, 15^ and may suggest that early abnormalities of lipid and glucose metabolism contribute to the contemporaneous increase in obesity and childhood asthma demonstrated in epidemiological studies.^23^ Modifications of early-life exposures prior to birth may impact on mechanisms leading to obesity and abnormal pulmonary L-arginine/NO metabolism.
